# Gene drive communication: exploring experts’ lived experience of metaphor use

**DOI:** 10.1080/14636778.2021.2020633

**Published:** 2022-01-12

**Authors:** Brigitte Nerlich, Aleksandra Stelmach

**Affiliations:** a School of Sociology and Social Policy, University of Nottingham, University Park, Nottingham, UK; b Business School, University of Exeter, Exeter, UK

**Keywords:** metaphors, gene drive, responsible science communication, public communication of science, stories in science communication

## Abstract

Metaphors have been crucial in making genetics and genomics public, from the code and the book of life to genetic scissors and gene surgery. A new field is emerging called “gene drive” – a range of controversial technologies that can potentially be used for the eradication or conservation of animal species. At the same time, metaphors are emerging to talk about the promises and dangers of “gene drive”. In this article we use thematic analysis to examine thirty interviews with gene drive science and communication experts, and stakeholders, focusing on how they talk about their lived experience of metaphor use in the context of gene drive communication, including their struggle to remember salient metaphors and their reflections on which metaphors to use and which to avoid. We discuss the significance of our findings for research and practice of responsible science communication.

## Introduction

Gene drive is a

genetic element that introduces a bias in the relative chance of inheritance between distinct versions of a set of genes, enabling one to spread rapidly in a population at the expense of others even if it is disadvantageous to the organism.

Gene drives occur naturally, but can now be synthesized, using gene editing, and can potentially “be exploited for the genetic modification of whole populations, such as disease-carrying insects” (Lexico 2020). These new genetic technologies have not yet been tested in the wild and controversies surround the potential consequences of doing so, which could be detrimental to human health and natural ecosystems (Brossard *et al*. 2019; Cobb forthcoming).

Unlike the term “gene editing”, which can be readily understood, at least superficially, “gene drive” is a rather obscure English term. This is slightly different in French, for example, where “forçage génétique” is more transparent, highlighting the forcing of a trait through a population. We shall focus here on English. As gene drive is an emerging field and the phrase itself obscure, one would normally expect a plethora of metaphors to emerge that attempt to make the unfamiliar familiar, with some of them becoming salient, such as “scissors” in the context of gene editing. Metaphors are crucial in the production and communication of knowledge in that “they allow us to make concrete connections between abstract concepts and everyday experiences” (Taylor and Dewsbury 2018, 1). This is especially important in science communication and particularly precarious when a field is just developing and trying to find its feet, theoretically and linguistically; and when communicators have to find their feet as well.

This article was prompted by emerging findings from a project investigating gene drive communication. We draw on 30 interviews with scientists, experts, and NGOs working in sectors or being involved in sectors relating to gene drive research in the United Kingdom, the United States, and Australia; we also undertook an analysis of traditional media articles about gene drive technologies published between 2015 and 2019 in the UK, the US and Australia. We were particularly interested in how the people we interviewed talked about gene drive, how they were told to talk about gene drive, and how they would recommend one should talk about gene drive.

We asked them about any analogies, metaphors or stories that they remembered and that they would recommend one might want to use in public engagement around gene drive, based on their interactions with others or with the media. The interviews followed on from the ethics approval for this research granted by Ethics Committee at the University of Exeter Business School (decision number 002986). All the participants provided informed consent and the interviews were recorded and later professionally transcribed.

The response to some of our questions was startling. Almost none of the participants remembered any metaphors or analogies when prompted to do so. Some remembered stories or themes. By contrast, when talking freely about their communication experiences, many participants used metaphors and engaged in reflections on their own or other people’s use of metaphors. This might not be that surprising, as finding examples of metaphors when put on the spot might be difficult, but it might also be indicative of both an absence of salient metaphors in gene drive discourse, and of a cautious approach towards using metaphors in public communication.

In this article, we want to explore what this struggle with metaphors may mean for science communication and public understanding of science. Before we do this, we provide some background to our analytical framework rooted in research on the role of metaphors in science and on responsible science communication.

## Metaphors in science

Metaphors are pervasive in everyday language but normally go unnoticed (Lakoff and Johnson 1980). When I say “You beat me in this debate”, I use the conceptual metaphor Arguments are war, for example. Metaphors can also be used more purposefully and deliberately, for example when people employ the conceptual metaphor Mask wearers are sheep in the current pandemic.

Metaphors are linked to myths and stories. They are indeed themselves compressed stories (Nemerov 1969). As early as 1644, Giambattista Vico (1948) pointed out that metaphors are like myths in miniature. In 1957 Roland Barthes noted in *Mythologies* (Barthes 1970) that myths are not just imaginative and “untrue” tales, but that we use them in everyday life to make sense of the world around us. In his book on modern myths like Frankenstein and Jurassic Park, the science writer Philip Ball has recently explored this sense-making in detail (Ball 2021). Through metaphors and narratives, we structure our views of the world and make it understandable.

Metaphors are also ubiquitous in science, as they allow scientists to capture new, unfamiliar, and abstract phenomena in terms of more concrete, familiar and more structured subject matters. For example, after the advent of printing, many philosophers and scientists started to think about nature as a “book” for example, a book that, now, after the advent of computers, can be more easily “edited”. It is therefore perhaps not so surprising that in

most biology (and science in general) concepts are actually metaphors. For example, biologists routinely refer to DNA as ‘book’; to DNA synthesis as ‘replication’; to RNA synthesis as ‘transcription’; to protein synthesis as ‘translation’; and to RNA modification as ‘editing’. (Kampourakis and Uller 2020, 102)

Building on a plethora of work on metaphors in science, most importantly perhaps by Dorothy Nelkin (1994, 2001) on popular and promotional metaphors, recent books by historians and philosophers of science Andrew Reynolds (2021) and Kostas Kampourakis (2021), have shown how essential metaphors are for every aspect of science, especially in the life sciences. They foster understanding (but also misunderstanding) of complex and/or novel issues by referring to concepts and objects from everyday experience, such as “genetic scissors” to describe what a new gene editing tool, CRISPR-Cas9, does to DNA.

However, science and technology studies scholars, as well as communication researchers, have rarely examined how scientists reflect on when, in the development of a field of science, metaphors are best used or deliberately introduced, and what responsible use may mean; which metaphors are helpful or unhelpful; and whether memorable or “sticky” metaphors are good or bad for science communication (but see Baake 2012; Nerlich and McLeod 2016; McLeod and Nerlich 2017; Sullivan-Clarke 2019). As Ball has pointed out: “The more vivid the image, the more dangerously seductive and resistant to change it is” (Ball 2011).

This article tries to find out empirically what and how experts think and talk about such issues in their daily work as scientists and communicators, and how, in the process, they grapple with responsible metaphor/language use. Our findings feed new insights into the emerging field of responsible science communication.

## Metaphors and responsibility

Since around 2009, when Andrew Balmer and Camille Herreman scrutinized the ethics of metaphor use in synthetic biology (Balmer and Herreman 2009), and Matthew Nisbet (2009) wrote about the ethical framing of science, reflections on responsible and/or ethical communication and metaphor use have taken off in the life sciences and the social sciences. In 2011, a synthetic biologist, Victor de Lorenzo, published an article entitled “Beware of metaphors: chasses and orthogonality in synthetic biology” (De Lorenzo 2011), followed by Massimo Pigliucci and Maarten Boudry (Pigliucci and Boudry 2011), Marianne Schark (2012), Eleanore Pauwels (2013), and Joachim Boldt (2018), all warning about the ethical and communicative pitfalls of machine or engineering metaphors in synthetic biology. Responsible science communication also began to flourish in the context of environmental science (Larson 2011; Kueffer and Larson 2014). Such reflections merged with a new trend in “responsible research and innovation” promoted since around 2011 (e.g. Owen, Macnaghten, and Stilgoe 2012).

When gene drive research began to accelerate in around 2015, it was therefore not astonishing that researchers were keenly aware of the need to not only carry out research responsibly but also to engage and communicate with the public responsibly, and ideally both together (e.g. Esvelt *et al*. 2014; Esvelt 2016; Long *et al*. 2020), especially with regard to a new technology like gene drive. As Matthew Cobb has pointed out in his forthcoming book *Genetic Dreams: The promises and perils of our most revolutionary technology* (Cobb forthcoming): “As scientists working on gene drives have recognised, sometimes it is right to be frightened by technology”. He quotes Austin Burt, one of the pioneers of “gene drive” (although he didn’t coin the term), as saying “Clearly, the technology described here is not to be used lightly. Given the suffering caused by some species, neither is it obviously one to be ignored” (Burt 2003, 927).

There is hence a communicative dilemma to be navigated responsibly, namely how to talk about an emerging and potentially controversial technology when it is still in the making, when it is still not clear how exactly it will work, and when it is still uncertain what the unintended consequences could be (see Nerlich and McLeod 2016; Kahn 2020). This dilemma extends to the decision about which metaphors – if any – should be deployed. As metaphors convey a certain image of technology, indeed a certain “truth” about its nature and possibilities (Brown 2003), the choice of metaphors, such as the “book of life” (Nelkin 1994), can promote and entrench positive or negative representations of science with important consequences for public understanding. Metaphors and the stories they tell can therefore have an ambivalent status. Though indispensable for conveying the nature of and the excitement about emerging fields, they can also misrepresent risks and benefits and cause exaggerated hopes and fears (Locke 2005; Davies 2008; Baake 2012; Taylor and Dewsbury 2018).

We can therefore see a trend emerging of reflections on responsible innovation and responsible communication across many disciplines. However, neither those studying responsible research and innovation, nor those analysing the ethical use of metaphors, nor those examining responsible science communication more broadly have as yet engaged with how gene drive practitioners themselves think and talk about the right or wrong, the timely or untimely, use of metaphors, including the unintentional forgetting and the intentional avoidance of metaphors in certain contexts or with certain audiences.

## Approach

This article was prompted by an observation made when reading interview transcripts in order to begin a larger thematic analysis and systematic coding. We noticed that when asked about memorable metaphors, most participants could not remember any. Despite that, most of them used metaphors copiously and discussed their pros and cons. This observation prompted our exploration of what one may call “metaphor talk” in the interviews, as a way of getting at a deeper understanding of our participants’ views, opinions, knowledge, and lived experiences regarding metaphor use in gene drive communication.

In order to study this metaphor talk, we used a focused form of thematic analysis. Qualitative thematic analysis has been described as “a method for identifying, analysing and reporting patterns (themes) within data” (Braun and Clarke 2006, 78). Amongst a variety of other patterns detected in a larger analysis, two patterns attracted our interest, namely the remembering and forgetting of metaphors, as well as the approving and rejecting of metaphors. These then guided our focused thematic analysis based on reading and re-reading those parts of the interview transcripts that dealt with metaphors, analogies and stories. After discussing emerging themes between the authors, we ended up with seven interrelated themes: forgetting metaphors; discussing overused metaphors; discussing controversial metaphors; reflecting on explanatory metaphors; debating helpful and unhelpful metaphors; talking about metaphors with multiple uses; getting to grips with the thorny issue of avoiding metaphors.

The focus in this article is therefore *not on analysing the metaphors used*, but on the way that *participants talked about metaphors*. The findings contribute insights into the particular difficulties encountered by experts trying to communicate gene drive and into issues related to (responsible) science communication more generally.

In the following, we first reveal some of the metaphorical, and mostly forgotten, origins of the phrase “gene drive”; we then briefly summarize what main metaphors we found in the media, before discussing the way that our interview participants talked about analogies, metaphors and stories relating to “gene drive”, those they forgot, those they remembered, those they found useful and those they did not.

## The metaphorical origins of gene drive

“Gene drive” is, as we pointed out, an obscure phrase. Where does it come from? Does it have a now forgotten metaphorical basis? Can that be used perhaps to communicate gene drive better, or, indeed not? The story of the creation of the term is a curious one (and would deserve a separate article).

While gene and genome editing have entered the *Oxford English Dictionary*, gene drive has, so far, not got its own entry. We found one hint at the origins of the phrase “gene drive” in a 2003 article by the popular science writer Oliver Morton for *New Scientist* entitled “Splat!” (Morton 2003). This article led us to one metaphorical source of “gene drive”. Morton reported on research carried out by Austin Burt at Imperial College London, especially on his 2003 article entitled “Site-specific selfish genes as tools for the control and genetic engineering of natural populations” (Burt 2003). But he also points out that

Burt is not the first person to consider messing around with mosquito genes in order to tackle malaria. Chris Curtis of the London School of Hygiene and Tropical Medicine, has been publishing on the subject since the late 1960s, and recently the field has been positively swarming with ideas.

Morton then relates a story told by Curtis, which sheds light on the metaphorical origins of the concept – we have highlighted aspects of the extended drive metaphor:

To solve this problem [spreading genes that make mosquitoes less likely to transmit malaria through a population at large], the resistance genes need to be *hitched to a ‘driver’* – a piece of DNA that spreads for some other reason. Various drivers have been discussed, including transposons and parasites that live within the mosquitoes’ cells, but they all share a significant drawback. ‘The crunch problem,’ says Curtis, ‘is how you make sure that the thing you want driven remains linked to the driving system.’ *If you think of the driver as a locomotive and the things you want driven as the carriages, he says, then if the coupling between them breaks, the locomotive will drive off into the distance while the carriages start to roll backwards.* There’s always a risk that a new mutation will *uncouple the driver and its carriages*, and even if the chances of this happening are very small, it’s still a fatal flaw. Work by some of Curtis’s colleagues suggests that if the engineered mosquitoes are just 20 per cent less fit than wild ones, and *even if the chance of uncoupling is as low as one in a million, the locomotive always runs away and the resistance genes die out*. To Curtis, that looks like the end of the line. ‘If we don’t have a reasonable prospect of *driving* those genes into wild populations, there’s no point.’

Nowadays, the “driving” element of gene drive is still transparent, as in “the gene is being ‘driven’ to progressively increase its frequency in the population” (Alphey *et al*. 2020), for example, but the etymological link to locomotives has been uncoupled, so to speak. It should also be pointed out that the word “drive” has been in use in biology since at least 1957 in research on “meiotic drives” (see Sandler and Novitski 1957) (Andrew Reynolds, p.c.), but this deeper history of the term would need a separate article.

In the following, we provide a brief overview of some metaphors used in the media and then go on to analyse the lived experience of metaphor use by experts in gene drive science and science communication in order to see how they talk about their experience with using gene drive metaphors.

## Metaphors in the media

The news media only started to report on gene drive issues around 2015, and the coverage was driven mainly by science-oriented press releases and some NGO protests. In the following, we can only provide some brief highlights.

Although the media articles provided a range of metaphors, and explanations of the gene drive technology (for discussion see Stelmach, Nerlich and Hartley Forthcoming; see also Kamenova, Akerman, and Emerson 2017), the original metaphor of a train/locomotive and carriages for gene drive was missing from the coverage. Nobody commented on what gene “drive” might actually mean. To explain “gene drive”, the press used a variety of metaphors, some fairly soberly explanatory ones, some more sensationalist explanatory ones and some falling back on entrenched metaphorical repertoires. These repertoires were grounded in well-established metaphorical descriptions of genes and genomes on the one hand (e.g. editing and scissors) and how to deal with epidemics on the other (e.g. war and battle), as gene drives can potentially be used to manage outbreaks of vector-borne diseases such as malaria or Zika.

Here are some examples. First, a sober explanatory metaphor, which is rather rare – based on the long-standing genetic metaphor of playing cards, well-known to Mendelian geneticists and sometimes called “the card game of life”: “gene drive technology works by forcing evolution’s hand, ensuring that an engineered trait is passed down to a higher proportion of offspring – across many generations – than would have occurred naturally” (*Agence France Presse* 05/12/2017). A more sensationalist, but still appropriate metaphor was: “From an evolutionary perspective a gene drive might be better regarded as a ‘gene bomb’: dropped into the normal course of inheritance, it annihilates natural variety” (*The Guardian* 10/06/2016).

Some metaphors were used (unconsciously perhaps) by scientists and then escaped into the wild and developed new connotations. This was the case for the “chain reaction” metaphor, especially the “mutagenic” chain reaction (Gantz *et al*. 2015). Chain reaction is a neutral scientific term – we all have now heard of PCR tests during the COVID19 pandemic – based on a polymerase chain reaction. However, out in the wild, the phrase “mutagenic chain reaction” developed connotations of nuclear chain reactions and merged with the “bomb” metaphor highlighting potentially catastrophic effects of gene drives. In his forthcoming book, *Genetic Dreams*, Cobb makes this associative link immediately: “According to the title of their [Gantz and Bier’s] article they had created a mutagenic chain reaction. To put it another way, they had made a genetic atom bomb”.

It was therefore not surprising to find other sensationalist metaphors in the media, such as: “Supercharged killer mosquitoes” (*Daily Star,* August 5, 2015). Some metaphors were more playful, focusing on the “drive” element of gene drive when journalists talked about “Dangerous driving” (*The Age, February* 24, 2018); “Darwin in reverse” (*The Washington Post, January* 6, 2016), and so on.

Unsurprisingly, we also found quite a few classic genomic metaphors, such as “Rewriting the code of life” (*The New Yorker,* January 2, 2017) and, of course, “Gene drive is an engineering technology in which the genetic code of a species is ‘edited’” (*The Weekend Australian Magazine*, May 26, 2018*)*.

In the context of disease and pest management, ubiquitous war metaphors dominated, as many talked about fighting, combating and battling insects, mammals and/or diseases; see this headline: “Mosquitoes, this time it's war; Zika is the last straw: Eradicate the world's deadliest creature” (*USA Today*, February 6, 2016). Sometimes battle metaphors also highlighted scientific disputes about gene drive themselves: “Scientists are sharply divided over using gene drives to eradicate pests, with Australia a battleground” (*The Age*, February 24, 2018).

And finally, a small number of journalists anchored gene drive reporting in some well-known stories about genetics, epidemics and invasive species, such as Jurassic Park, the Sorcerer’s Apprentice or Faust.

## Interview analysis

Given that gene drives have deep metaphorical origins and attracted at least some metaphorical framing in the media, what metaphors do people working on the topic of gene drive remember when they talk about gene drives and how do they communicate about gene drives in interviews? In the following, we will discuss some of the answers provided by our interview participants which we have thematically sorted into seven metaphor-focused themes.

### Unmemorable metaphors

When reading through the interviews, it was startling to find one standard reply to us asking participants whether they remembered any metaphors used in discourses about gene drive in the media or elsewhere. One phrasing of such an answer is prototypical: “I can’t”, “can’t think of any right now” (UK01); “I can’t think of any off the cuff” (UK4); “There aren’t that many … maybe I’m not remembering. I can’t think of many people using analogies or any” … (UK06). “I don’t know, I can’t think of anything off the top of my head. It’s a good question though” (USA17). “I don’t know. That’s a good question. None immediately come to mind”. (USA18) etc. There may be multiple reasons for this memory loss, such as being put on the spot, not having thought about metaphors before and so on. As one participant said: “If there were [any metaphors] I would probably already have used them” (UK04). Remarkably, we found that most of the participants, even those who said that they do not remember any metaphors, used metaphors in their interviews with us. This reflects our earlier observation that people might use metaphors unreflectively much more often then they think, but it might also be that there are just no salient metaphors of gene drive around yet that would trickle into general conversation about this technology.

### Memorable myths and metaphors

Some participants resorted to memories of the most lurid genetic and genomic myths and metaphors which have been used for a long time, since at least the 1990s with relation to genetic modification:

you always see things like Franken whatevers, Franken chickens or Franken, that sort of thing, anything to, I guess, raise the interest in people (AU27)

there’s always this Franken-mosquito, which has … I think it’d be interesting from your analysis, but I think it’s disappearing. There’s a bit less. At the beginning there were a lot of that, if I remember. It’s not based on data; it’s just my memory, so I might not be as sensitive any more or whatever, but I feel this was more at the beginning, this kind of Franken-mosquito, and the kind of image is coming from science fiction, the whole *Jurassic Park*, it’s something that people … I don’t know, they resonate. (UK06)

Others mentioned the story of Dr Strangelove (AU29) or the Sorcerer’s Apprentice, which they regarded, unlike Frankenstein and even Jurassic Park, as rather appropriate: “One of the stories which I feel is very much in place is that of The Sorcerer’s Apprentice. I feel like that is a very, very apt story to go with it.” (UK09) The sorcerer’s apprentice is a story about someone who is unable to contain or control a situation, event, or process that they instigated – it becomes a runaway event like a gene drive.

Stories seem to be more memorable than metaphors, including the tips of their narrative icebergs, namely their titles which have become cultural icons in debates about genetics and genomics, such as Frankenstein (Turney 1998; Ball 2021).

### Overused metaphors

On the whole, then, participants did not remember metaphors immediately, but they remembered some stories. However, when asked about how they talked about gene drive, rather than asking about metaphors, analogies and stories, some participants reflected on metaphors, old and new, as well as on the shortcomings of using metaphors, analogies and stories.

One participant stressed the importance of discussing gene drive technologies in the context of the history of biotechnologies, such as recombinant DNA. They recommended that we try and go beyond overused metaphors, such as playing God, that we don’t use the same old metaphors and images we have used since the 1970s and recombinant DNA over and over again, and, most importantly that we “place this particular technology in the broader history of biotechnology so that we understand where the technology comes from, we understand its limitations, how it builds on existing forms of technical practice” (UK05).

But how do we get to better metaphors and more novel metaphors? Which metaphors are suitable for discussing gene drive and which ones are not? And does this depend on the cultural context in which the metaphors are used?

### Controversial metaphors

One participant reflected on the dangers of a rather controversial metaphor, that of birth control, as gene drives can be used to make target species infertile (Callaway 2015):

I can’t think of many people using analogies or any … Sometimes some people use the idea of birth control. (…) I think it’s rare and I think it’s a good thing it’s rare because it’s a very problematic issue in Africa and in many other countries as well. (UK06)

Other participants used the (equally controversial) analogies of vaccination (USA11) but also the “sterile insect technique” (USA19), which they regard as less controversial. Both birth control and vaccination can have negative connotations in certain socio-political and cultural contexts and therefore need to be navigated carefully as source domains for metaphors.

Some participants remembered the metaphor of “chain reaction” (see Gantz *et al*. 2015), but thought that using it to frame gene drive was “terrifying language”:

Chain reaction [laugh] goes back to nuclear reaction where a chain reaction occurs and is unstoppable and is dangerous. So a less confusing, more neutral term would have been good but it’s too late. It’s gone. It’s out of the box. (USA14)

By contrast, other participants thought the “nuclear bomb” metaphor was actually quite appropriate and one should use it to talk about the risks of gene drive technologies (UK07). None of the participants endorsed the metaphor of gene drive as a “weapon of mass destruction” and no one used war metaphors.

Some participants thought it might be better to talk instead about Darwinian evolution, despite evolution being a controversial topic in some parts of the world. A number of participants, like many scientists working in this field, used the metaphor of “selfish genetic element” without explanation (USA11) or reflection, as it has been a common jargon term since the 1960s (Enzmann 2018). They warned however against using the metaphor of “hijacking evolution” which they found was over-hyped and too “click-baity” (USA14), and therefore might create negative perceptions. So what metaphors were used when trying to explain gene drive?

### Explanatory metaphors

Many participants talked about metaphors which have become salient and ubiquitous in the context of explaining CRISPR and gene editing. And as CRISPR is relevant to modern gene drive technologies, some of these metaphors migrated into the gene drive discourse. There is, of course, the pervasive metaphor of “molecular scissors” used by this participant for example: “I tend to start from the beginning of just saying what we want to do and then I explain molecular scissors and I explain what we want to do”. (UK06)

Another important metaphor, also used in epigenetics (Stelmach and Nerlich 2015) is that of “switches”, as in this quote recommending that one should provide safety devices for gene drives. It is argued that to not turn into runaway trains, so to speak, gene drives need, in a way, responsible innovation.

When we just talk about a gene drive, people think about it as a runaway drive, one that we can’t get back, but in reality what we really want to deploy are gene drives that have safety switches in them that would have either a limit in space on where they would spread or a limit in time in terms of how long they would drive for. (AU22)

Only once did a participant use one of the most apt explanatory metaphors, which was also strangely absent from most of the media coverage and the memory of our participants, namely that of tossing a coin:

I usually say things like with most genes it’s like tossing a coin, head or tails, there’s a 50% chance that you’ll pass it onto the next generation, whereas with gene drive what we’re trying to do is to change that 50/50 ratio and instead ensure that we’ll always get a head or we’ll always get a tail. In other words, we’re always going to pass on a particular gene to the next generation. What that means is then that that gene would spread through the population because it’s always been passed on. (USA11)

### Unhelpful and helpful metaphors and stories

While the coin toss metaphor, although rarely used by our participants and never remembered, is rather helpful when trying to explain what gene drive is or does, one participant pointed out that the original drive metaphor was surely not helpful and warned about potential connotations, stressing that once you create a metaphor and analogy and it is out there you are stuck with it but you can’t control it – it is as “run-away” as gene drives.

Metaphorical uses of ‘drive’, I find people just get tripped up in how they use it … , all this talk about things, doesn’t necessary work well, but like I said, they’re stuck with it because it’s the term they’ve started with. Yeah, I do think it’s interesting. It’s one of those things where you really wish people had perhaps asked a science communicator, a rhetorician of science or somebody before they tagged it with this. ‘What is this gonna connote? How are people gonna understand it? What’s the gap in the analogy that you’ve implicitly developed here?’ It might still have been a useful term, but I think it’s not as helpful as it could be. (AU25)

In an ideal world, which is not always possible to achieve when communicating emerging sciences, metaphors should be used thoughtfully and reflexively, weighing up how helpful or harmful they may be. They should be communicative “technologies of humility” rather than hubris (Jasanoff 2007). However, in the quick flow of research, competition and communication this reflective use of metaphors is not always possible (Radford 2009).

If, as the participant pointed out, some metaphors, such as gene drive itself, are so unhelpful, what about helpful metaphors or analogies? Are there any at all and why are they needed? Some participants pointed out that metaphors or analogies are needed in a situation where one is talking about something completely new:

most people won't have a clue what you’re talking about, so you would need to use analogies to take the essence of the science idea and put it in a context that people will understand. (AU22)

One science communicator stressed that it might be useful to shock people a bit to create awareness of a new technology, by using an extreme metaphor, like, indeed, “atomic bomb” to highlight the potential of the runaway nature of gene drives – especially given that it might not be a “metaphor” at all but just a good description of what happens with the release of a gene drive: “ … I would say it’s a valid way of getting over the rapidity and the uncontrollability of the gene drive, which is basically just exponential expansion” (UK07). But using the word bomb is not just descriptive; it is emotive too:

it’s there to shock, it’s done deliberately. Nobody has ever complained, I’ve given this as a talk to scientists as well, and none of them ever said no, that’s not a fair metaphor. It’s not a precise metaphor but the point is there to deliberately shock and to get over the idea of the uncontrollability and the runaway nature potentially. (UK07)

The participant pointed out that this can be used as a preamble to talking about various ways to regulate new technologies, comparing gene drive regulation for example to air flight regulation.

Importantly, the expert noted that using a scare metaphor alone is not enough. It is important to stress that scientists have been discussing the pros and cons, risks and benefits of gene drives for a long time: “I also emphasize that the scientists themselves are very concerned about this and that the starting point for much of the alarm has come from the scientists who are working on it.” (UK07)

This participant tells people a whole story in which they embed one shocking metaphor. This shocking metaphor links to many other storylines and themes attached to gene drive, in this case, the history of regulation of technologies, such as civilian air flight and nuclear power (UK07).

Participants remembered in particular stories which stressed the dangers of gene drives, such as messing with nature (UK08), eradication, extinction and annihilation. Some participants cautioned against evoking “extinction” stories as gene drives don’t necessarily cause extinction (USA17). They suggested that communicators don’t overpromise but also don’t overhype and instead talk honestly about potential risks and threats, as well as benefits (USA12). They thought it might be good to stress the lifesaving potential of the technology and the avoidance of the death of children caused by malaria (USA20). They recommended that one compares the regulation and control of gene drive technologies with that of other technologies and stress the importance of the precautionary principle for example (UK08), instead of focusing on catastrophic stories of ecosystems collapsing – “it’s just sort of creating this catastrophic risk that doesn’t really exist, an impression, a feeling of that” (USA17).

Metaphors are therefore not good or bad, helpful or unhelpful per se but they are put to good or bad use in a context and according to a certain intention. And finally, we should keep this advice from one of the participants in mind:

I don’t think there’s a magic bullet, there’s a metaphor, to find a way to explain it and I’m not sure that you really need to anymore than people really need to understand about CRISPR beyond the fact that the gene editing, pair of scissors, cut and paste metaphor, I think that does it fine. (UK07)

This is quite an optimistic view of metaphors. More pessimistic ones were also expressed with relation to confusing metaphors that have multiple uses, and the avoidance of metaphors altogether.

### Multiple use metaphors

One participant, after some prompting, mentioned a few metaphors, amongst them the “Trojan Horse” metaphor, but pointed out that

My reaction is not necessarily negative, because they’re trying to give some sort of easy-to-understand message that goes, in some sense, to how they work, at least. The problem is that some of these terminologies can be taken in a negative sense and imply deceit or malfeasance. (UK03)

This comment highlights a real problem with the Trojan Horse metaphor and needs a bit of unpacking. As Nerlich (2020) has pointed out, in the context of gene drive, the Trojan Horse metaphor can have two uses, an explanatory one and an inflammatory one. The explanatory use can be exemplified by the following extract from an ethical review of genome editing by the Nuffield Council on Bioethics: “it may be possible to use a vector (e.g. a virus) as a kind of Trojan Horse to introduce genome editing tools to make the necessary repairs within the patient’s body”. (Nuffield Council on Bioethics 2018, 41)

On the other hand, as, for example, reported in the *New York Times* at the time of the interviews, the Trojan Horse metaphor can also be used by opponents to gene drive technologies to argue that the ultimate aim of introducing gene drive technologies was corporate profit for agriculture (see Kahn 2020). This means that one metaphor can have two quite different uses and meanings, can lead to confusion and might therefore be avoided.

### Avoiding metaphors

Some participants explicitly reflected on the potential impact their metaphors and analogies might have, for good or for ill, and that it might therefore be prudent to avoid such comparisons:

I remember once comparing it and feeling really bad, because it’s something we had said we wouldn’t do, just because you’re never sure of the analogy, you’re never sure what it carries, all of these things, so you’d have to really study the analogy. (…) So we’ve never really used it, and I remember once using it (…) and being like mortified that it would become the thing (…) (UK06)

One participant pointed out that this might be a reason why they couldn’t remember a metaphor or analogy in the first place!

P: Mm [pause]. I can’t really think of anything [pause]. We always try to explain it in quite … without using too many analogies or too many stories so that people don’t mix … so it’s very hard for my brain to think of one now.

I: Oh, that’s very interesting. You’re trying not to use stories and not to use analogies?

P: Yes (UK06)

The participant went on to elaborate that they always try to explain gene drive without using analogies, at least not too many, and that this might have contributed to their not remembering any. Using metaphors and analogies was for them dangerous, as one cannot control their connotations, which could potentially skew the ways in which the audience understands the scientific issue at hand (see Davies 2008).

### Advice on metaphors

Given the participants’ difficulties in recalling memorable metaphors, the problem of using or avoiding metaphors and the general confusion over which metaphors might be useful and which not, some participants asked for help from the social scientists with which they were in conversation.

One Australian participant said that they would actually like to know what metaphors, analogies and stories were being used and whether they were any good: “I can’t think of any but if somebody tells you one, can you let me know? … I wish I could help but I’d actually like to know the answer to that question.” (AU21) As a US participant quipped: “I mean I don’t know; that’s an empirical question.” (USA14)

Given the problems associated with finding and using “good” metaphors relating to gene drive, participants pointed out that it would be helpful to have more research on this topic, especially in the current situation where gene drive research was still complicated and uncertain.

I imagine they’re probably are some good metaphors, I just don’t know what they are. […] And so I would say that precision in language is really important when you’re talking about something that is as complicated and still as uncertain as gene drive is. (USA15)

Others stressed that it is not about finding the best metaphor to explain things to people – any adequate metaphor will do; and it’s not about

settling on consistent terminology, I mean, I think we should give it up and recognise that gene drive means self-propagating and we should come up with different terms for the others […] like self-exhausting for things like daisy drive, that it should spread for a while and then run out of gas and stop. (USA12)

This and other participants also suggested that, instead of standardizing terminology (see for example Alphey *et al*. 2020), the focus should be on effective storytelling and their relevance for people’s experience (see Davies 2008), as science thrives on stories, and as metaphors are key ingredients in such stories.

Communicating gene drive is thus a rather complex task, which involves remembering, finding, selecting and relating stories and metaphors that are suitable to the task at hand and to the audience involved in the conversation.

## Discussion and conclusion

In this article, we set out to explore the way some experts talk about gene drive with a focus on their lived experience of metaphor use, including not remembering metaphors they do indeed use themselves. This is a first study of such lived experience of metaphor use which provides new insights into the way experts negotiate gene drive communication, including trying to use metaphors responsibly.

In our analysis, we found that when prompted to remember metaphors and analogies of gene drive, interview participants were thoroughly perplexed and nonplussed, and they struggled to remember any salient metaphors. This may be because recalling metaphors may be difficult in general, or because, quite often, participants try to not use metaphors themselves. In addition, gene drive has, as yet, not provoked a wide public debate which would draw on striking or salient metaphors, like cloning or DNA for example, where certain images and metaphors became ubiquitous and iconic, such as the double helix or the metaphor of the “book of life” (see Nelkin and Lindee 1995). This means that the participants do not yet have at their disposal a repertoire of obvious, tried-and-tested metaphors to be used to communicate gene drive.

However, despite this potential difficulty, when talking about their communication experiences in general, some participants mentioned metaphors, analogies and stories and even discussed their pros and cons, that is, they showed a general awareness of responsible metaphor use. Some of these metaphors overlapped with metaphors we found in the media, mostly metaphors linked to older and better-known topics and technologies, such as evolution, Mendelian genetics, gene editing and so on.

It should also be stressed that what lay readers would regard as metaphors may not be seen as such by experts, such as “selfish genetic element” or “biased inheritance” or even “gene drive” itself, which means that such terms would not be “remembered” as metaphors.

Some entrenched metaphors, like “molecular scissors”, popular in gene editing discourse, that reach from the lab to the popular press, were used (and remembered) in gene drive communication. The card game or coin tossing metaphors which could, in principle, play an important role in science communication were not remembered and not used, it seems, for reasons that are not self-evident, while the original “drive” metaphor was regarded as too obscure to be used for science communication and mainly forgotten.

There may be two reasons why metaphors had such an ambivalent status in our participants’ discourse. On the one hand, there may be, as yet, no salient and memorable metaphors available with which to explain and communicate gene drive. On the other hand, participants may have adopted a rather cautious approach to metaphor use, an approach also identified in other areas of science communication (Davies 2008; Baake 2012).

For the participants in our study communicating gene drive was often perceived as risky or even dangerous (Davies 2008), because, in the absence of standardized ways of talking about it, it could mislead lay publics and misrepresent the technology. On the other hand, communicating and engaging with lay publics was seen as an important component of working in a scientific or science-related field. As a result, the participants seemed to have developed an awareness that what was said about gene drive mattered and could shape public perceptions of this field. This led some to carefully consider which words, metaphors or stories were controversial or acceptable, helpful or unhelpful, and to express the view that empirical studies about metaphor use would help them resolve this communication dilemma. The difficulty of talking about this new field even prompted some others to avoid metaphors and analogies altogether, thus restricting their means of explaining this technology to lay publics. In that, they implicitly followed Philip Ball’s advice who said in 2011: “Maybe we are too eager to find a neat metaphor rather than just explain what is going on as clearly and honestly as we can.” (Ball 2011) By contrast, a minority of participants were willing to embrace the use of metaphors and stories, even controversial ones such as “a gene bomb”, in order to spark interest and make gene drive relevant for lay audience (see Davies 2008).

This ambivalent status of metaphors is a sign that “gene drive” as a field is still grappling with the question of how language should be used in public debates about this technology (see Alphey *et al*. 2020; Schairer, Triplett, and Buchman 2020; MacDonald, Edwards, and Balanovic 2021). Communicating about, and engaging others with, a new science in these circumstances is very difficult. Using one of the most well-established tools for science communication, metaphor, becomes challenging. Our interview participants were highly aware of these challenges, highlighted some of them and pointed to ways of dealing with them – all aspects of responsible communication that were clearly on their minds. We have come a long way from the 1990s, when Nelkin and others criticized the promotional use of metaphors in genetics and genomics research to attract attention and public support. Our novel findings regarding the lived experience of gene drive communication and the use of metaphors should stimulate further research into responsible science communication with a new focus on practitioner perspectives.

Expert practitioners were aware of the severe limitations of the original drive metaphor; they were skeptical of over-hyped evolution as well as eradication metaphors; they advocated embedding metaphors, if used at all, in precautionary and responsible narratives; and they also wanted future research to study the use and misuse, risks and benefits of metaphor use and avoidance empirically, so that public engagement efforts could be improved based on evidence. There is more work to be done. Science communication is first and foremost a practice, and we need to listen to the voice of practitioners when trying to fathom how to communicate gene drive in general and how to communicate gene drive responsibly in particular.
